# Assessing clinical relevance of off-target effects observed at suprapharmacological concentrations of SGLT2 inhibitors. Reply.

**DOI:** 10.1172/JCI211778

**Published:** 2026-10-01

**Authors:** Abdualrahman Mohammed Abdualkader, Rami Al Batran

**Affiliations:** Faculty of Pharmacy, Université de Montréal, Montréal, Quebec, Canada.

**Keywords:** Endocrinology, Metabolism, Chronic kidney disease, Diabetes

**The authors reply:** We thank Dr. Taylor for his commentary (1) and welcome the opportunity to clarify the scope of our conclusions (2). Our central finding — that sodium-glucose cotransporter-2 inhibitors (SGLT2i) directly activate 3-hydroxy-3-methylglutaryl-CoA synthase 2 (HMGCS2) — is supported by convergent in vivo, genetic, cellular, and cell-free biochemical evidence, together with structural modeling. We address his specific points below.

Dr. Taylor correctly notes that the 100 μM concentration of empagliflozin used in human hepatocytes and the 3 mM concentration used in the drug affinity responsive target stability (DARTS) assay exceeds estimated free-plasma concentrations achieved with approved human dosing. Our DARTS experiment used a high ligand concentration to maximize detectable protease protection in a highly competitive and complex whole-lysate environment and was intended to support target identification, not to define clinical potency (3). The purified-enzyme experiments similarly establish that empagliflozin can activate human HMGCS2 under the tested conditions, not the magnitude of target engagement achieved in patients. We note, however, that a free-plasma comparison may underestimate hepatic exposure. Empagliflozin is a substrate of the hepatic uptake transporters OATP1B1 and OATP1B3 (4), and a nonclinical review identified the liver among the tissues with the highest empagliflozin concentrations after oral dosing (5). Hepatic partitioning has been reported for other SGLT2i; for example, enavogliflozin exhibits a 5.8-fold liver/plasma AUC ratio in mice (6). Most directly, empagliflozin dose-dependently increased HMGCS2 activity and β-hydroxybutyrate (βOHB) in our fasted mice. Berger et al. reported that 50 mg/kg/day administered in the diet produced a peak plasma concentration comparable with published human exposure data (7). Their chronic dietary dosing supports exposure at 50 mg/kg but not equivalence with our acute protocol; direct measurement of plasma and unbound hepatic empagliflozin is needed to define exposure more precisely.

Our conclusion nonetheless rests on convergent evidence rather than any single experiment. Empagliflozin increased the activity of purified recombinant human HMGCS2 in a cell-free system containing no SGLT2, and three structurally distinct SGLT2i produced similar effects. Enzyme kinetics, molecular docking, molecular dynamics, and phosphomutant studies further supported allosteric modulation of a catalytically favorable HMGCS2 conformation (2). Activation was also observed in intact human hepatocytes, demonstrating that the response extends to human liver cells.

We did not propose that allosteric HMGCS2 activation explains diabetic ketoacidosis (DKA). Comparing our approximately 25% change in enzyme activity with the greater than 10-fold increase in ketonemia during DKA conflates distinct physiological settings and assumes a direct relationship between an enzyme assay and circulating metabolite concentration. Our study identified an additional hepatic mechanism that may contribute, alongside the established substrate-driven pathway, to the modest ketone elevation observed in patients receiving approved SGLT2i doses. It does not address the severe ketogenesis driven by insulin deficiency and unrestrained lipolysis in DKA. HMGCS2 catalyzes the first committed, rate-limiting step of ketogenesis; therefore, even a fractional increase in HMGCS2 activity may influence ketogenic flux when accompanied by sufficient substrate availability.

Our 5-minute experiment showed that hepatic HMGCS2 activity and βOHB increased in mice before glycosuria-driven metabolic changes were detectable. This finding does not establish that ketonemia generally precedes glucosuria in patients, where dosing, absorption, and metabolic context differ substantially from our acute protocol. Empagliflozin did not increase βOHB or HMGCS2 activity in fed mice, indicating that the acute response depends on metabolic state and is consistent with substrate availability constraining ketogenesis. However, we did not directly manipulate substrate availability or measure target binding in the fed state. The rapid mechanism identified here may complement, rather than replace, the established effects of chronic glycosuria on substrate mobilization and ketogenesis.

We agree that the absence of the SGLT2i-induced increase in hepatic HMGCS2 activity and βOHB in liver-specific Hmgcs2-deficient mice establishes that hepatic HMGCS2 is required for this ketogenic response but does not, by itself, prove that direct activation of HMGCS2 mediates this response). This genetic evidence should be interpreted together with the cell-free activation, kinetic, structural, and cellular findings. Collectively, these orthogonal approaches support direct biochemical modulation of HMGCS2 under the conditions tested.

In summary, our findings establish HMGCS2 as a direct biochemical target of SGLT2i under the tested conditions. The quantitative contribution of this mechanism at approved human exposures remains an important question requiring measurement of intrahepatic drug exposure, target engagement, and ketone production flux in clinically relevant experimental systems and, ultimately, patients.

## Version 1 10/01/2026

Electronic publication
